# Training community resource center and clinic personnel to prompt patients in listing questions for doctors: Follow-up interviews about barriers and facilitators to the implementation of consultation planning

**DOI:** 10.1186/1748-5908-3-6

**Published:** 2008-01-31

**Authors:** Jeffrey Belkora, Brian Edlow, Caryn Aviv, Karen Sepucha, Laura Esserman

**Affiliations:** 1 Department of Surgery, University of California, San Francisco, San Francisco, USA; 2 School of Medicine, University of Pennsylvania, Philadelphia, USA; 3 Center for Judaic Studies, University of Denver, 2 Denver, USA; 4 Health Decision Research Unit, Massachusetts General Hospital, Boston, USA

## Abstract

**Background:**

Visit preparation interventions help patients prepare to meet with a medical provider. Systematic reviews have found some positive effects, but there are no reports describing implementation experiences. Consultation Planning (CP) is a visit preparation technique in which a trained coach or facilitator elicits and documents patient questions for an upcoming medical appointment. We integrated CP into a university breast cancer clinic beginning in 1998. Representatives of other organizations expressed interest in CP, so we invited them to training workshops in 2000, 2001, and 2002.

**Objectives:**

In order to learn from experience and generate hypotheses, we asked: 1) How many trainees implemented CP? 2) What facilitated implementation? 3) How have trainees, patients, physicians, and administrative leaders of implementing organizations reacted to CP? 4) What were the barriers to implementation?

**Methods:**

We attempted to contact 32 trainees and scheduled follow-up, semi-structured, audio-recorded telephone interviews with 18. We analyzed quantitative data by tabulating frequencies and qualitative data by coding transcripts and identifying themes.

**Results:**

Trainees came from two different types of organizations, clinics (which provide medical care) versus resource centers (which provide patient support services but not medical care). We found that: 1) Fourteen of 21 respondents, from five of eight resource centers, implemented CP. Four of the five implementing resource centers were rural. 2) Implementers identified the championing of CP by an internal staff member as a critical success factor. 3) Implementers reported that modified CP has been productive. 4) Four respondents, from two resource centers and two clinics, did not implement CP, reporting resource limitations or conflicting priorities as the critical barriers.

**Conclusion:**

CP training workshops have been associated with subsequent CP implementations at resource centers but not clinics. We hypothesize that CP workshops combined with an internal champion and adequate program resources may be sufficient for some patient support organizations to implement CP.

## Background

People diagnosed with a serious illness such as cancer usually face complex decisions regarding treatment. Visit preparation interventions are designed to help patients get ready to discuss treatment decisions with their providers. Visit preparation interventions range from low-cost prompt sheets and proformas to more intensive preparatory sessions that include coaching. Studies have found visit preparation to have modestly positive effects, such as improving the number and quality of questions asked, especially about sensitive topics such as prognosis [1-12]. A systematic review suggests that the early evidence about this new class of interventions consists of 'a series of tantalizing but disconnected and unconfirmed results.' The authors conclude that visit preparation may be worth implementing for other reasons [13]:

'In terms of practice there are strong justifications unrelated to evidence-based medicine for adopting a collaborative approach to the medical encounter, such as, for example, patient preferences and moral imperatives.'

Another recent systematic review, 'Interventions before consultations for helping patients address their information needs,' concurs about the modest positive effects of visit preparation interventions, and then states:

'Despite these apparent benefits, we know of no routine implementation of strategies to help patients address their information needs' [14].

Our team has been associated with a routinely implemented form of visit preparation, an intervention called consultation planning (CP), which has been in use at a university breast cancer center since 1998. One of the authors (JB) developed CP as part of his doctoral research, advised by one of the present co-authors (LE) and assisted by another (KS) [15]. In CP, a trained facilitator or consultation planner helps newly diagnosed patients brainstorm and write down questions and concerns for their doctor [16]. The consultation planner uses a prompt sheet or template (see Appendix 1 for the most recent edition) to survey the patient for questions and concerns, and then documents these in a consultation plan, or patient agenda for the upcoming visit. Copies of the consultation plan are printed out for the patient, family members, and physicians to use as a visual aid during the appointment. (See Appendix 2, a real case with all patient identifiers modified or suppressed.) Consultation planners are trained not to provide advice or information, but rather to focus on eliciting and documenting patient questions and concerns.

Based on our studies of CP showing reduced communication barriers and enhanced patient and physician satisfaction [17,18], and other studies showing benefits of visit preparation [1-14], we integrated CP into routine clinical care at the University of California, San Francisco (UCSF) Breast Care Center in 1999. Since then, the service has been offered free of charge to newly diagnosed patients thanks to government and foundation grants as well as faculty discretionary funds. We have previously published reports on our UCSF experience [19-21].

In 2000, 2001, and 2002, we responded to *ad hoc*, word-of-mouth expressions of interest in CP by individuals affiliated with resource centers and clinics in our region. For the purposes of this report, we define our region as the nine counties of the San Francisco Bay Area plus two North Coast Counties (Mendocino and Humboldt), comprising over seven million people and over 14,000 square miles. We define clinics as organizations that provide medical services to patients in exchange for fees or private or public insurance reimbursements. We define resource centers as organizations that provide non-medical supportive services (such as information and emotional support) at no financial cost to patients, financed either by charitable contributions or by budgetary contributions from a parent organization such as a medical center.

We opened our internal workshops, conducted annually to train personnel at our university breast cancer center, to all self-referred individuals who heard about the training through informal networking among regional clinics and resource centers. The trainees' organizations paid for print materials, transportation, meals, and lodging while our institution, UCSF, donated the space and instructor time.

The CP training workshops included lectures, structured role playing, and group discussion sessions. The training handouts included templates, checklists, and reference materials summarizing lecture topics. It is important to note that consultation planners are trained to avoid providing medical advice or information. Rather, they learn how to elicit, paraphrase, summarize, and document patient questions and concerns in accordance with our SCOPED model of decision making [22].

The significance of the present report is that while evidence is suggestive about the effectiveness of visit preparation in academic settings, and there are ethical and patient-centered reasons to implement visit preparation, little is known about efforts to disseminate such interventions. We sought to learn whether our training workshops were leading to uptake of CP in our region, and if so, learn more about the implementation experience.

## Methods

### Design

We conducted follow-up interviews with people who attended one of three training workshops intended to facilitate implementation of the CP visit preparation technique. We sought and obtained ethics approval from the UCSF Institutional Review Board.

### Aims and questions

Our aim was to follow up with trainees from three workshops, learn about their post-training experiences, and generate hypotheses about whether and how to promote implementation of CP. Our inquiry addresses the following questions:

1) How many trainees implemented CP?

2) What facilitated implementation?

3) How have stakeholders (trainees, patients, physicians, organizations) reacted to CP?

4) What were barriers to implementation?

### Setting, population, sample, and recruitment

Our team reviewed records of training workshops held in October 2000, April 2001, and April 2002, and one of us (BE) attempted to contact 32 local trainees by phone to recruit them for interviews. Twenty-six of the 32 local trainees came from eight resource centers while six came from four community clinics around our region.

If a trainee agreed to participate, our interview recruiter (BE) scheduled a phone appointment and sent a consent form and an interview guide via fax, e-mail, or postal service. The recruiter attempted to contact non-respondents a total of three times.

Respondents were also asked to refer the recruiter to physicians who were known to have seen patients who had engaged in CP. Through this snowball referral, the recruiter identified four surgeons and two oncologists and contacted them for interviews. Due to privacy concerns and resource limitations, we did not contact patients.

### Data collection

The interview recruiter (BE) also conducted interviews based on semi-structured interview guides, one for trainees and one for physicians. Another author (CA) supervised the interviewer's draft of the interview guides, which were reviewed and revised by other members of the team (JB, KS, LE). Both interview guides assessed whether the respondents had implemented CP (trainees) or seen CP patients (physicians), and probed for general reactions as well as indications of implementation facilitators and barriers. The interviewer conducted interviews between 13 December 2002 and 18 March 2003.

### Analysis

At the end of data collection, the interviewer (BE) abstracted and tabulated quantitative or descriptive variables of interest to us in addressing the questions. Then, both the interviewer and his supervisor (CA) read the transcripts and engaged in thematic coding [23,24]. After discussion, they established consensus categories, assigning each a color. Then one of the data analysts (BE) went through the transcripts line by line, assigned the appropriate color(s) to phrases that fit the themes, and generated summary reports of the phrases assigned to each theme. Both data analysts (CA and BE) then discussed and agreed upon the creation of subcategories within each theme. One of the data analysts (BE) re-coded the transcripts using the finalized themes and sub-categories, noting cases where respondent reports deviated from the proposed themes and subcategories. As a result of this process, two themes were modified to accommodate opposing views of the same dynamic (see Results – resource limitation and leadership attention themes). All members of our team then discussed the analysis, which was documented in a chronologically ordered audit trail of printed materials, and participated in the drafting and revision of this report.

## Results

### Respondents

**Figure 1:**
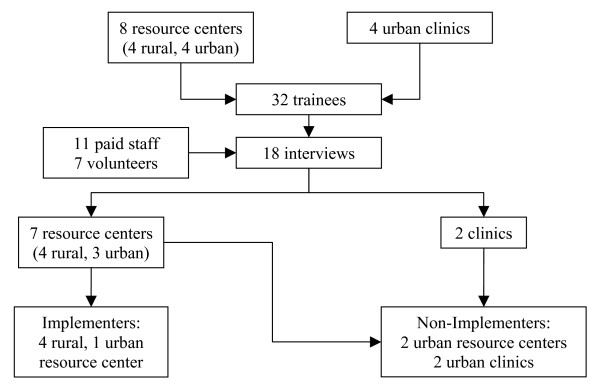
**Study flow chart**. This flow chart shows the population and sample of organizations and individuals represented in the training workshops and interviews, respectively, and indicates the number that implemented or did not implement the Consultation Planning intervention.

Fourteen trainees declined or did not respond after three attempts to reach them, leaving 18 of the 32 trainees who agreed to be interviewed. These 18 trainees represented seven out of eight community resource centers and two out of four community clinics that had sent staff or volunteers to CP training sessions held at UCSF in October 2000, April 2001, and April 2002 (Figure 1).

Of the 18 trainee respondents, 14 were women and four were men. Eleven participants were paid staff, including three executive directors, while seven were unpaid volunteers. Four of the participants were under 40 years old, while 14 were between ages 40 and 65.

One surgeon and one oncologist declined to participate, saying they were too busy, leaving three surgeons and one oncologist. Of the four physician respondents, two were women and two were men. All had been referred by rural community resource center trainees.

### How many trainees implemented CP?

Fourteen of the 18 trainee respondents were providing CP at their organizations, seven as unpaid volunteers (Table 1). They were associated with five of the ten organizations represented among the respondents, all five of which were resource centers (out of seven resource centers in the sample and eight in the population of trainees). In other words, only but not all resource centers had gone on to implement CP. The 14 implementing trainees collectively reported performing 203 CP interventions between 8 October 2000 and 10 March 2003. The remaining seven respondents, representing two clinics (out of four) and two resource centers, did not implement the CP intervention (Figure 1).

**Table 1:** Selected performance characteristics of implementing resource centers

	# of Consultation Planners Trained	# of consultation plans	# of Years Since Training
Resource Center #1	2	67	2
Resource Center #2	1	12	1
Resource Center #3	3	21	1
Resource Center #4	2	32	2
Resource Center #5	5	71	3

**Total (mean)**	**13 (2.6)**	**203 (40.6)**	**(1.8)**

This table shows the number of trainees, number of consultation plans, and years since training for resource centers that implemented Consultation Planning

### What facilitated implementation?

Interview respondents consistently identified the presence of an internal champion in their organization as the most important determinant of their ability to engage in CP with patients. Respondents from the implementing resource centers consistently commented that there was one staff member in each organization identified with CP, and who invested time and energy in shepherding its implementation. The five internal champions were all paid employees, and three were the administrative leaders of their organizations.

One self-identified internal champion explained, 'The program would not run if there wasn't someone actively committed to making it run and referring patients.... Out of all our programs, CP is probably the one that requires the most time and organization to make it work...and I have done whatever I can to keep the program afloat.'

Similarly, an internal champion at another organization coordinated referrals, trained additional volunteers, and gave up her office to other consultation planners so that they could conduct private CP sessions when no other rooms were available. One internal champion, who was also an administrative leader, wrote a grant to fund her own full-time position as consultation planner and to provide all the resources necessary for program implementation.

### How have stakeholders (trainees, patients, physicians, organizations) reacted to CP?

#### Trainee views of interactions with patients

Two major themes emerged in the trainee interviews related to delivering the intervention to patients: the sense of trainee satisfaction due to the perceived value of the CP intervention to patients; and the adaptations made by trainees as they implemented CP.

Indeed, all implementing trainees volunteered that providing the CP intervention was a rewarding experience. The prevailing sentiment was that the intervention provided a much-needed service to patients. One consultation planner asserted that the intervention mitigated the patients' confusion with the decision-making process because 'all of a sudden it's not this big mess with no beginning and no end – it's an orderly list of questions.' Another consultation planner noted, 'it's amazing how much patients say they've gained clarity, because I'm not adding anything new. I'm just organizing what was already in their heads.' According to one consultation planner, 'the relaxed nature of the CP session gives patients the opportunity to tell me what's going on with their life. The doctor doesn't have time to extract these important issues. So, because of my work, the doctor can learn key things about a patient in five minutes [by reading the consultation plan] that I've learned in an hour and a half.'

Regarding local adaptation of CP, at least one trainee at each of the five implementing organizations had provided the intervention to non-English speaking patients, through the intermediary of a professional translator or by having a family member translate for the patient. Two organizations have established collaborative relationships with local social service providers focused on the needs of Spanish-speaking people, in order to assure the availability of free, professional translation services to Spanish speaking patients who desire to engage in CP.

#### Physician reactions to CP

By virtue of the snowball referral scheme, the four rural physicians interviewed had all experienced consultations with patients who had engaged in CP. The major theme that emerged from physician interviews was their perception of how CP leads to more productive consultations. As a sign of their endorsement of CP, three of the four interviewed physicians routinely referred patients to their local resource center for CP, while the fourth was in the process of establishing a referral process. The physicians felt that CP facilitated patient-physician communication. One physician explained that when a patient brings a consultation plan to an appointment, 'I am ecstatic because I know the patient has already thought about her options and is more organized.' Another physician stated that patients who use the intervention 'are definitely more prepared – they have a clear agenda, and they handle the decision-making process better.' Other representative comments included, ' [CP] helps patients organize their thoughts,' and 'the patients tend to ask different, more sophisticated questions. Instead of starting at square zero, the patient has already dissected the information down to a second or third level.'

#### Organizational leadership attention to CP

According to the trainee interviews, three of the five implementing resource centers had the explicit support of their Boards of Directors, including the executive directors. One Board made CP an organizational priority because its members believed that CP was their 'most marketable tool' and that the appeal of the intervention would lead to higher patient utilization of other support services. One trainee explained, 'the Board likes CP because it's a tangible tool that people can wrap their brains around. [Patients think] 'oh yeah, they can do that for me.'' The Board of Directors at another organization regularly discussed the CP program at quarterly meetings and invited consultation planners to share their experiences.

By contrast, two other implementing organizations reported little, if any, attention from the organizational leadership. One trainee reported, 'we have not gotten specific financial or logistical support.... They said, 'yeah, it's a good program, implement it,' but they didn't help us with the implementation... Before I went to the training, there was no planning discussion as to implementation strategies; and there has been no such discussion post-training.'

#### Barriers to implementation

Two main themes emerged from the interviews regarding the barriers to implementation: resource limitations; and lack of patient access to health care.

#### The resource limitation barrier

Trainees from the three clinics and two resource centers that did not implement CP identified a variety of implementation barriers. The most commonly mentioned barrier, cited by trainees from all five non-implementing organizations, was resource limitations.

This conclusion reflected two different underlying dynamics. The first dynamic was that CP was a new service and would require new resources because existing programs addressed higher priority needs in the community: 'It's a whole new program. [Implementation] would require us to write a grant...for training additional staff.' One trainee explained, 'for our organization, we've found that paying for diagnostic care like biopsies and mammograms is more of a need for uninsured women.' Another respondent said: 'We don't have the space. We don't have the money. And yet we think this is a great idea.'

On the other hand, one trainee from a non-implementing organization believed that the visit preparation service offered by consultation planners was too similar to existing types of support: 'we set up patients with patient navigators, so to try not to duplicate services, we're holding off with [CP] right now.'

#### The patient access barrier

Poor patient access to health care was another barrier cited by two respondents at non-implementing organizations. They felt it would be futile to offer CP services to patients who were not actively or successfully engaged with the health care system. One trainee reported, 'it is idealistic to think that physicians would continually refer their patients to us for CP. It speaks to economic class, because most of our patients don't have access to their doctors for more than five minutes.' The other respondent (from a different organization) concurred that for her population of low-income, underserved women, 'it is difficult to try to facilitate a seamless flow of information [via CP] when there is not a seamless flow in [patient] access.' These respondents also believed that physicians were not willing or able to engage patients in the decision-making process, thereby making it futile to attempt to implement visit preparation interventions.

## Discussion

### Main findings

A primary finding of our interviews was that 14 of 18 respondents did implement CP after only a single day of training, and with no other technical assistance from CP developers and trainers. This represents a lower bound of 14 out of 32 trainees, assuming that none of the non-respondents implemented CP. All of the implementing trainees were affiliated with community-based resource centers, four of which were rural and one urban. According to trainees from implementing organizations, the main factor that promoted successful implementation was an internal champion on the paid staff who was dedicated to program implementation.

Implementing trainees reported positive effects of CP for themselves, patients, and physicians. Physicians corroborated this view of CP as a beneficial service. Trainees revealed that they have expanded CP beyond the scope of training to include medical translators. Physicians had established referral agreements with resource centers in order to ensure that patients engaged in CP sessions before their medical visits.

Conversely, four out of 18 responding trainees – from two community clinics and two resource centers, all urban settings – did not implement CP. According to these trainees, the main barriers to implementation included the scarcity of program resources and the need to prioritize services more basic than CP, in some cases because CP was realistically suited to the context in which they operated (*e.g., *underserved patients without access to care, much less high degrees of involvement in consultations.) We found a split between trainees from organizations that would implement CP given additional resources, and those that were frustrated at the assumption, which they saw as inherent in CP, that patients already had access to care.

### What this work adds to the existing literature

The present report focuses on dissemination through training workshops of our CP techniques for helping patients prepare a list of questions before their medical visits. Our report extends the body of studies related to the efficacy of visit preparation in academic settings, as reviewed in the Background section. This report, which focuses on visit preparation by non-medical personnel, also complements a few emerging accounts of training workshops related to the provision of decision support by medical providers. Our interview data suggest that a short training workshop can stimulate implementation of CP, which is consistent with the early findings of other implementation studies in closely related areas.

For example, Légaré and colleagues taught shared decision making skills to 120 physicians in a brief interactive workshop [25-28]. Among other findings, the workshop improved the degree to which physicians' post-visit decisional conflict agreed with that of their patients [25]. The authors concluded that 'just one interactive workshop of 1.5 hours with feedback and a reminder at the point of care might be sufficient to influence the agreement between patients' and their physicians' perception of the decision-making process.'

Stacey and colleagues used a three-hour online tutorial combined with a three-hour follow-on workshop to teach decision support skills to call-center nurses [29-31]. The training was found in a randomized controlled trial to promote better decision coaching skills. In a later implementation case study, 11 of 25 nurses trained in a decision support protocol through an online tutorial, workshops, and performance feedback, were actively using the protocol. The remainder indicated they had not yet had a case suitable for the protocol. Twenty-three of the nurses indicated they would use the protocol in future cases requiring decision support.

### Methodological strengths and weaknesses

Our aims in conducting post-training interviews were more oriented towards hypothesis generation than extensive description or causal explanation. Our inquiry had some strengths. This is the first report of training non-medical personnel to engage patients in visit preparation. We followed up with a group of potential intervention adopters who had sought out training and we were able to identify and interview implementers. And our questions and analyses revealed definite patterns for further investigation, such as our finding that only resource centers, and no clinics, implemented the CP intervention.

However, several limitations should be noted in interpreting this report on our interviews. While we were focused on hypothesis generation, others might be tempted to interpret interview findings from a quantitative or positivistic philosophical perspective, in which case we must point out biases in our data. Our interviews suffer from two levels of selection bias, meaning that the respondents are likely to differ systematically from non-respondents and others. First, trainees in our workshops had sought us out to learn more about the CP intervention. Our results may not generalize to less motivated individuals and organizations. Second, there were 18 respondents and 14 non-respondents, and the respondents reflected more implementers (14) than non-implementers (4). This may be the result of a propensity for implementers to participate in this kind of follow-up interview to a greater degree than non-implementers. Had we reached the remaining 14 trainees, who were likely to be non-implementers, findings within our sample might reflect different barriers to implementation, and a less positive view of the reactions to CP.

Regarding reactions to CP (question three in our aims), due to privacy concerns and resource limitations, we did not interview patients or physicians (other than those referred by trainees), or administrators. Instead, we prompted interview respondents for their firsthand reactions to CP, as well as their secondhand and anecdotal accounts of patient, physician, and organizational leadership reactions to CP. Therefore, our report suffers from measurement bias, and this portion of our report should not be interpreted as a report on the effectiveness or impact of CP. For hypothesis generation purposes, however, we felt that it was valuable to characterize the respondents' perceptions of how CP implementation was received by others.

Although we sought disconfirming evidence and deviant cases in eliciting and analyzing the interview data, our own status as developers of the intervention, and providers of the training workshops may have created additional measurement bias.

In addition, our data were gathered retrospectively rather than prospectively. Between one and three years had passed since the training workshops by the time we interviewed trainees. This introduced another possible measurement bias, due to inaccurate recall.

Regarding the qualitative side of our inquiry, as for any such analysis we struck a balance between the scope, quality, resources, and time taken to analyze our data. Our focus on hypothesis generation and our limited resources means we could justifiably be criticized for not doing more inter-rater reliability checks (*e.g., *more multiple coding of transcripts), more triangulation of findings (*e.g., *reviewing other records and doing observations in addition to interviews), and more respondent validation [32].

### Implications for practice, research and policy

The aims of our inquiry were oriented towards hypothesis generation rather than analytic generalizations. Based our findings, we have generated a hypothesis that CP workshops combined with an internal champion and adequate program resources may be sufficient conditions for some organizations to implement CP.

Additional questions stimulated by our interviews include whether the needs of rural communities are better suited to CP implementation than those of urban communities; and whether community resource centers are better suited than community clinics to delivering CP. The existence of referral agreements between physicians and resource centers reinforces the finding that clinics may not be the best channel for delivering CP, but may be open to partnering with resource centers to make sure their patients get the service.

A related hypothesis emerging from our interviews is that CP may be most suitable in settings where navigation or other programs assure support for patient access to care, at which point improving patient participation in medical consultations is an appropriate goal.

The presence of seven satisfied and productive volunteers among the implementing respondents suggests that delivery models combining professionals and volunteers may be one way to address the barrier of resource limitations.

Although our inquiry was not suited to determine impact or effectiveness of CP, the anecdotal evidence we collected suggests that CP may be as well-received in some community settings as it and other forms of visit preparation have been in academic settings. Indeed, since conducting these interviews, we have also reviewed survey records at two of the resource center sites and discovered that satisfaction with CP there is very high [16].

The findings of our interviews have led us to pursue collaborative projects with three of the highest-volume CP service providers in our sample: the Cancer Resource Center and Woman's Cancer Advocacy Network in Mendocino County (which have merged since the completion of our interviews), and the Humboldt Community Breast Health Project in Humboldt County, California. In 2003, a pilot study involving these community organizations and UCSF was funded by the California Breast Cancer Research Program to adapt CP for broader use across these two rural, underserved counties, with special attention to serving Native American Indian and Spanish-speaking minorities. We are also providing technical assistance to another resource center, the Community Breast Health Project of Palo Alto, CA.

## Conclusion

This report adds important information about the potential for training programs to disseminate visit preparation interventions that, like CP, incorporate a significant coaching component. We found that five organizations were able to implement CP on the basis of our one-day training program, with positive effects reported by 14 trainees and four physicians. Four community resource centers in rural settings were among the implementers. Critical success factors included the presence of a motivated and effective internal champion who could count on adequate programmatic resources. Non-implementers did not have the resources to add CP and could not incorporate it into existing funded programs. Some non-implementers questioned the appropriateness of CP for patients that lacked access to care. Based on the findings of our interviews, and the questions and hypotheses that have been raised, the developers of CP have embarked on a program of community-based research with rural resource centers.

## Competing interests

The author(s) declare that they have no competing interests.

## Authors' contributions

BE, CA, and LE conceived the follow-up interviews and invited JB and KS to participate in their design and coordination. BE and CA collected the data and performed the qualitative data analysis. JB led all authors in writing and revising the manuscript. JB and KS conceived of and designed the tables and figures. All authors read and approved the final manuscript.

## Appendices

### Appendix 1. Prompt sheet for consultation planning

Trainees are taught to use these probes to prompt patients to articulate their questions and concerns prior to meeting with a doctor.

#### Situation

What do you know about your situation? Questions about key facts? Diagnosis? Test reports? Pathology report? Anything unusual?

#### Choices

What can you do? Questions about tests? Active monitoring (no further treatment)? Treatment options? Second opinions? Clinical trials? Complementary therapies? Newest treatments? Most proven treatments? Most aggressive treatments? Least aggressive? Middle ground treatments? Remedies for side effects? What to stop doing? What to start? Decisions to make now? Decisions to make later? Past decisions to revisit?

#### Objectives

What do you want? Goals for doctor's appointment? Goals for treatment? Preferences about length and quality of life? Regarding quality of life: what to continue/protect? (*e.g., *relationships, work, hobbies, daily activities, body image, sexuality, child-rearing, etc.)? Preferences about timing, frequency, duration, intensity, location, costs of treatment? Concerns about interactions with other treatments or medical conditions? Hopes? Fears? Unspoken thoughts or feelings? Learning preference: visual or auditory or other type of learner? Preferred approach to decision-making: holistic or analytical? Qualitative, informal (*e.g., *talking about pros and cons, journaling/writing)? Quantitative, formal (*e.g., *rating and weighting? Statistical number-crunching)? Somewhere in between? (*e.g., *filling out a table)? If quantitative: interested in survival/recurrence/complication rates for each choice? Prefer rates to be explained in numbers (*e.g., *60% ten-year survival rate) or words (*e.g., *more likely than not to survive)? Level of effort to expend in analyzing decision? Resources?

#### People

Who can help? Questions about where else to go for advice or information or support? How do you want this doctor involved in your treatment decisions? Other doctors? Other people (*e.g., *who come to appointments)? Whom do you want to have a voice in analyzing your decisions (i.e., seek their input)? A vote (involve them in arriving at a final decision)? Visibility (keep them informed)? Who else do you need to talk to? Anyone to exclude?

#### Evaluation

How does each choice affect each objective? Questions about specific choices and specific objectives? Baseline prognosis (prognosis with no further treatment)? How choices will affect survival, recurrence (*e.g., *rates for patients like you?) How choices will affect quality of life? Likelihood of complications, short and long-term side effects? Best-case scenario, worst case, most likely (*e.g., *in terms of survival, quality of life) for each choice?

#### Decisions

Which choice is best? What are the next steps? Who will do what, when, where, why, how? What resources can help overcome any barriers to next steps? If undecided/unready: Timeline/deadline for arriving at a decision? Priority relative to other commitments? Resources for gathering information/data about how choices affect objectives? Who can help (revisit People above)? What do you want (revisit Objectives above)?

### Appendix 2 – Sample consultation plan (de-identified)

This Consultation Plan, based on a real case but stripped of identifying details, shows an example of the questions and concerns that a Consultation Planning trainee elicited from a breast cancer patient before her meeting with her oncologist.

#### Situation

After the second excision, is the cancer in the margins gone?

#### Choices

Are there better tests than mammograms? I would like to know other options, such as sonograms, and how often I could receive additional testing.

#### Radiation

Tamoxifen – how long would I have to take it?

Letrozole? (had bad muscle pain)

#### Objectives

I feel very strongly about preventing a recurrence. I am not afraid of death, but am afraid of pain and suffering.

I want to know whether to take Tamoxifen now.

I used to go to the Senior Center, but I have not gone since I got the cancer.

#### People

How often will I see Dr. Oncologist?

#### Evaluation

What is my prognosis? What is the percentage for recurrence after having Stage II Cancer?

What is my prognosis with Tamoxifen? I know a lot of people that have taken Tamoxifen and have survived for many years.

Tamoxifen – side effects?

Do I need to exercise and go on a special diet?

When I took Letrozole, I felt very fatigued and could barely walk. Will Tamoxifen cause the same side effects? I am currently taking Lipitor for cholesterol and Hydrocorothiazide for high blood pressure. I also take Advil for pain. Can I continue taking these medications while taking Tamoxifen?

#### Decisions

I will make the decision with my doctor's advice.

I will make a decision today on Tamoxifen
